# Study based on bibliometric analysis: potential research trends in fluid management for sepsis

**DOI:** 10.3389/fmed.2024.1492396

**Published:** 2025-01-10

**Authors:** Sihan Liu, Haoting Pei, Jing Wang, Lujun Qiao, Hao Wang

**Affiliations:** ^1^Department of Critical Care Medicine, Qilu Hospital, Shandong University, Qingdao, China; ^2^Innovation Research Center for Sepsis and Multiple Organ Injury, Shandong University, Qingdao, China; ^3^School of Nursing and Rehabilitation, Shandong University, Jinan, China; ^4^Shengli Oilfield Central Hospital, Dongying, China

**Keywords:** sepsis, fluid management, bibliometric, R software package, VOSviewer, CiteSpace, hotspots

## Abstract

**Objective:**

To investigate the potential and evolving trends in fluid management for patients with sepsis, utilizing a bibliometric approach.

**Methods:**

Scholarly articles pertaining to fluid therapy for sepsis patients were extracted from the Web of Science (WoS) database as of June 1, 2024. The R software package, “Bibliometrix,” was utilized to scrutinize the primary bibliometric attributes and to construct a three-field plot to illustrate the relationships among institutions, nations, and keywords. The VOSviewer tool was employed for author analysis, keyword co-occurrence analysis, and data visualization. Additionally, CiteSpace was used to calculate citation bursts and keywords.

**Results:**

A comprehensive retrieval from the Web of Science (WoS) database yielded a total of 2,569 publications. The majority of these articles were predominantly published by two countries, namely the United States (US) and China. Among the myriad of journals, Critical Care and Journal for Intensive Care Medicine emerged as the most prolific. In terms of institutional contribution, the University of California System stood out as the most productive. Recent analysis of keywords revealed a significant citation burst for terms such as “balanced crystalloids” and “critically ill children”.

**Conclusion:**

There is a growing focus on the connection between fluid management and the treatment of sepsis, with research in this area being at an advanced stage.

## Introduction

Sepsis is a life-threatening organ dysfunction caused by a dysregulated host response to infection (1). This definition highlights, on one hand, the mechanisms and severity of organ dysfunction triggered by infection, while on the other hand, it reveals the importance of timely recognition of sepsis and the need for early intervention and clinical treatment by healthcare professionals (2).

Currently, sepsis remains a major healthcare burden worldwide, including in both high-income and low- to middle-income countries. It has become one of the significant public health issues globally, posing a tremendous threat to the safety of millions of lives each year and can lead to up to 25% (or even more) of in-hospital patient deaths (3). According to World Health Organization statistics, approximately 189 deaths occur among every 100,000 hospitalized sepsis patients (4). In the United States, deaths caused or triggered by sepsis account for 1/3 to 1/2 of all in-hospital deaths (5). A study by Fleischmann et al. estimates that the global annual incidence of sepsis could be as high as about 31 million cases, with an estimated 19.4 million cases of severe sepsis and around 5.3 million deaths (6). Another study by Jawad et al., examining sepsis incidence and mortality rates across multiple countries, similarly indicates that in developed countries, the mortality rate of sepsis ranges from 40% to 50%, while septic shock can be as high as 80% (7).

Early administration of antibiotics for infection control is one of the core components of sepsis treatment. In cases of sepsis or septic shock, every hour of delayed targeted antimicrobial therapy significantly increases mortality, with an average increase of 7.6% (8). In addition to antibiotics and source control of infection, fluid resuscitation is a fundamental therapy for sepsis. Effective early fluid resuscitation in septic shock is crucial for stabilizing tissue hypoperfusion induced by sepsis.

Fluid therapy can improve tissue perfusion by increasing stroke volume through volume expansion, and it is an important component of clinical treatment for sepsis (3). The therapeutic effects of different fluids vary, and they can even impact patient prognosis (9). Insufficient fluid therapy could potentially result in diminished cardiac output in patients, thereby leading to a decrease in the effective circulating blood volume and insufficient perfusion of essential organs. Conversely, an overabundance of fluid therapy may precipitate circulatory overload, cardiac failure, and tissue edema. Empirical evidence suggests that maintaining a negative fluid balance during the treatment of sepsis can enhance patient prognosis (10). Therefore, precise volume management for patients with sepsis presents a significant challenge in clinical practice (11).

Currently, there is a substantial amount of literature reporting on treatment strategies and guidelines for fluid management in sepsis (12, 13). Effective fluid management is significant for improving patient prognosis, enhancing quality of life, and reducing the burden of disease (14). The management of septic shock poses significant complexities due to the intricate nature of the pathological alterations in the circulatory system, which are not easily rectified through singular fluid therapy approaches. Although numerous fluid management paradigms exist, none have achieved comprehensive endorsement. A conspicuous absence of objective assessment and synthesis of scholarly literature from scientific research databases underscores the necessity for a systematic examination and review of extant studies.

Bibliometric analysis originated in the UK in the 1920s and is the best choice for providing detailed trends of research activities in a specific field over time (15). This type of analysis has produced numerous research outcomes in disciplines such as library science, biomedicine, technology management, and engineering management (16). Bibliometrics is an academic discipline that scrutinizes the attributes of literature systems and bibliometric characteristics, executing both quantitative and qualitative evaluations of published works, including books and scholarly articles. Beyond delineating and forecasting the progression of particular research domains, this analytical approach can also juxtapose the contributions made by distinct countries, institutions, academic journals, and scholars (17).

Information visualization constitutes an interactive, analytical methodology that augments data representation through the utilization of computer graphics and image processing techniques. This technique is capable of intuitively exhibiting the intricate and abstract semantics concealed within voluminous datasets, typically in the form of knowledge graphs. The said approach boasts advantages in unveiling the extensive scope and profound depth of information resources, thereby providing superior time and cost efficiency.

This study comprehensively analyzes the current status of fluid management in sepsis patients based on the Web of Science (WoS). Utilizing bibliometric methods, this research aims to uncover research trends in this field and predict potential future research hotspots.

## Materials and methods

### Data sources and search strategy

Web of Science (WoS) was utilized as the primary database for conducting bibliometric analysis, widely accepted for its comprehensive coverage. A comprehensive online search of literature from 1978 to 2024 was performed on June 1, 2024. The search strategy included terms: TS = [sepsis OR (severe sepsis) OR (septic shock)] AND TS = (fluid management) AND Language = English. Publication types were limited to articles, excluding retractions and book chapters. The search results were exported in plain.txt format for further analysis, complete records and cited references also included (18). A total of 2,569 publications on the topics of sepsis and fluid management were identified.

### Bibliometric analysis

Bibliometrix, an R package, was employed for quantitative analysis. Key extraction fields included: authors from the AU field (affiliations from AU*_UN field and countries from AU_*CO field), publication years from the PY field, keywords from the DE field, and citations from the TC field. Version 4.0.0 of Bibliometrix was utilized to quantify publication counts, citation frequencies, compute keyword usage, assess collaboration strength among countries/authors, and construct a tri-field map for keyword analysis.

VOSviewer was used to visualize keyword networks related to sepsis and fluid management research. Through co-occurrence analysis, keywords were clustered and colored based on temporal evolution using Average Appearance Year (AAY) to quantify relative novelty (19).

CiteSpace, a web-based Java application, was employed for data analysis and visualization (20). Leveraging co-citation analysis and pathway network scaling, it generated visual maps to explore development trends in a specific field of literature. This study utilized CiteSpace to identify highly cited bursts of publications or keywords within defined periods (21). This article utilizes CiteSpace software to identify widely cited literature/keywords with strong citation bursts over a specific period. The study divides the time frame into three stages: 1978–1994, 1995–2009, and 2010–2024, for the analysis of keyword bursts over a duration of 15 years.

## Results

### Analysis of annual publication output

**Figure 1:**
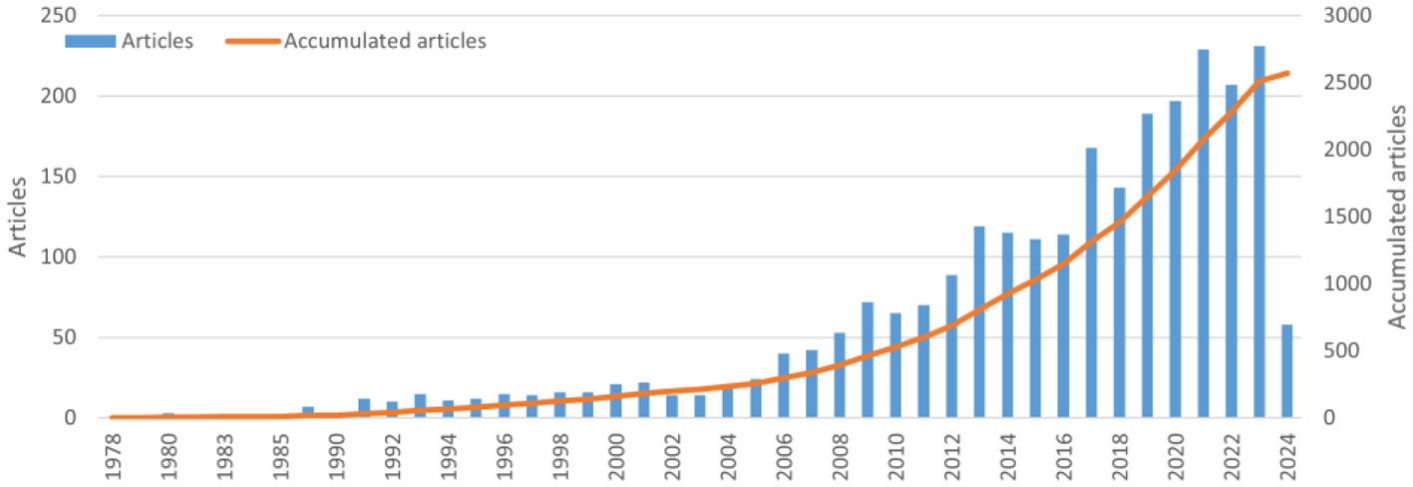
The annual number and the cumulative number of publications.

Between 1978 and June 1, 2024, a total of 2,569 publications on the topics of sepsis and fluid management were identified, spanning 46 years. Figure 1 illustrates the annual and cumulative publication counts related to sepsis and fluid management literature. From 1978 to 2006, the cumulative number of publications steadily increased from 1 to 297. Subsequently, from 2007 to 2023, there was a rapid growth in publication output, culminating in a cumulative total of 2,569 publications by June 2024.

### Analysis of national publication volume and collaboration

**Figure 2:**
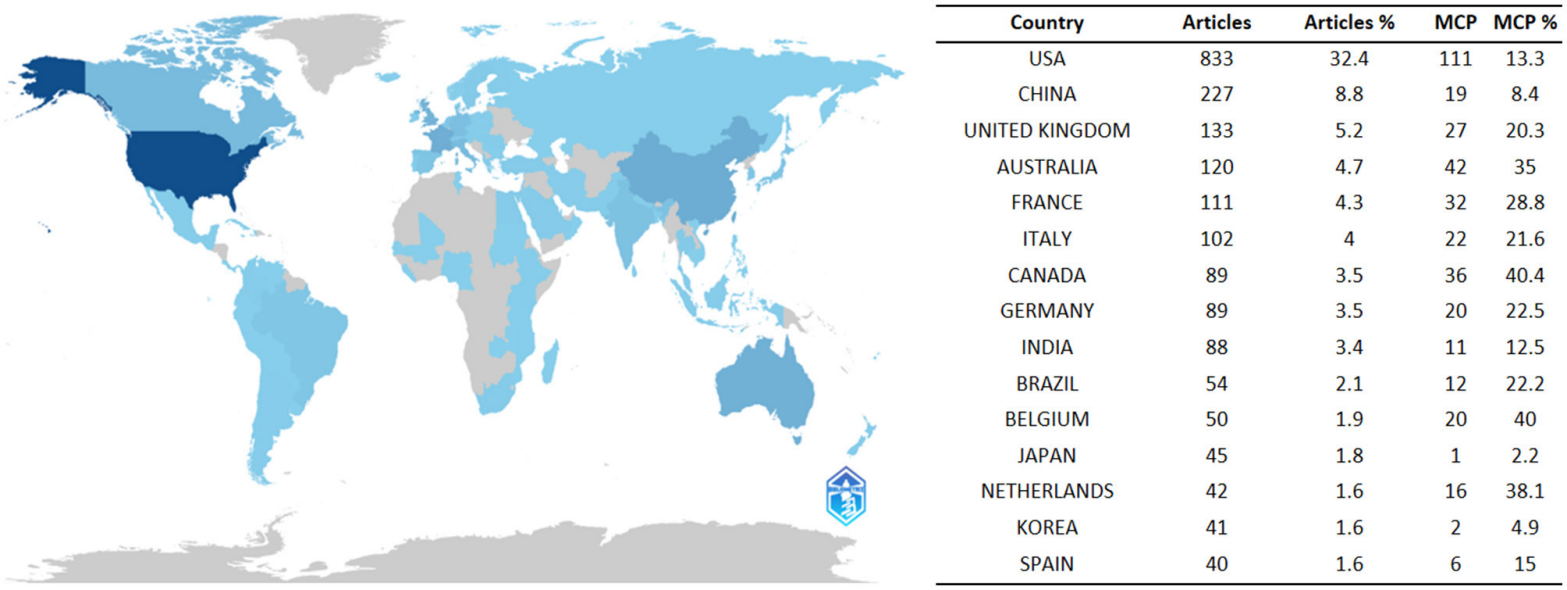
A map of country contribution based on the article output.

An analysis of the number of publications by each country reveals that articles from 76 countries/regions were identified in the relevant field. As shown in Figure 2, the United States had the highest number of published papers (*n* = 833), accounting for 32.4%. China ranked second (*n* = 227, 8.8%), followed by the United Kingdom (*n* = 133, 5.2%), and Australia closely behind (*n* = 120, 4.7%). Other countries in the top 15 published more than 40 papers each.

Multiple Country Publications (MCP) indicate the number of publications involving co-authors from different countries/regions. While the United States had the highest MCP count (*n* = 87), its MCP ratio (=MCP/articles) was only 13.3%. Australia ranked second in MCP count (*n* = 42) with a ratio of 35%, followed by Italy (*n* = 36) with a ratio of 40.4%.

### Analysis of institutional output and collaboration

**Figure 3:**
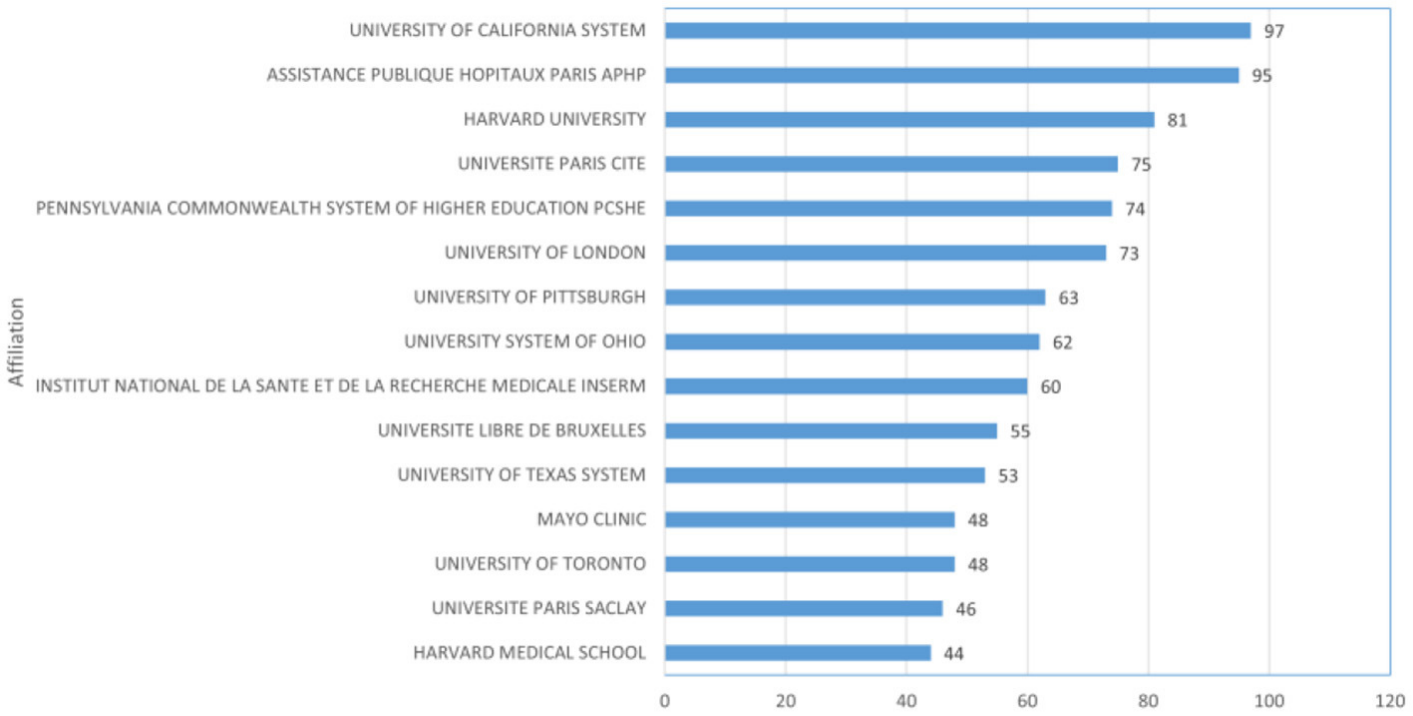
The top 15 institutions with the most publications.

In total, 3,432 institutions conducted research related to sepsis and fluid therapy. The top 15 institutions are listed in Figure 3, with the University of California (UNIVERSITY OF CALIFORNIA SYSTEM) leading with 97 publications.

**Figure 4:**
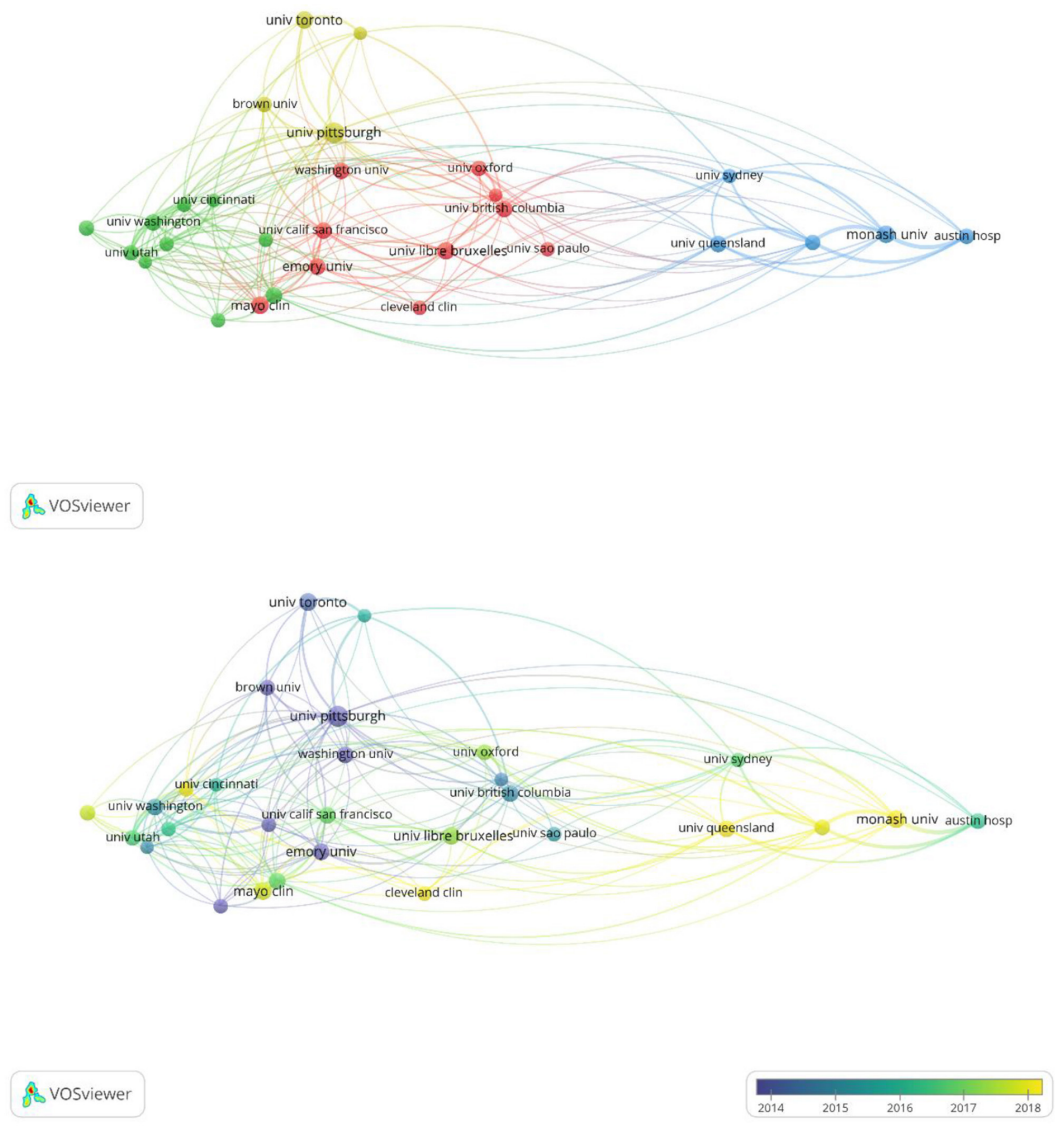
Clustering network for the co-authorship analysis.

Furthermore, we undertook an analysis of collaborative authors in order to examine inter-institutional relationships. In the clustered network analysis of collaborative authorship, node size corresponds to the quantity of publications produced by each institution, while node colors signify clusters distinguished by levels of collaboration intensity. Within Figure 4, a total of 29 institutions were segregated into four distinct clusters, with the largest cluster (highlighted in red) consisting of 10 institutions.

In the analysis of the collaborative author network within the field of sepsis and fluid management, colors are utilized to represent the average publication years of each institution. Harvard University and Washington University WUSTL are noted as early pioneers in this area, while researchers from Université Libre de Bruxelles and the University of Pittsburgh have demonstrated significant activity in recent years specifically in fluid management for septic patients.

### Analysis of article output and impact of journals

A total of 867 journals were identified as publishers of the 1,030 articles analyzed in this study. Table 1 presents the top 11 journals based on article output, including their most recent Impact Factors (IF). Leading the list is Critical Care from England, with 96 articles published, followed closely by the Journal of Intensive Care Medicine from the United States, which published 80 articles and demonstrated significant influence. Of these top journals, 4 are ranked in the first quartile (Q1) of the Journal Citation Reports (JCRc1).

**Table 1:** Top 11 journals with most articles about tumor burden and immunotherapy.

**Rank**	**Journals**	**Articles**	**Country**	**IF**	**JCRc**
1	Critical Care	96	ENGLAND	15.1	Q1
2	Critical Care Medicine	80	US	8.8	Q1
3	Journal of Critical Care	47	US	3.7	Q3
4	Shock	45	US	3.1	Q3
5	Intensive Care Medicine	42	US	38.9	Q1
6	Current Opinion in Critical Care	40	US	3.3	Q3
7	Cureus Journal of Medical Science	39	N/A	1.2	N/A
8	American Journal of Emergency Medicine	37	US	3.6	Q3
9	PLoS ONE	35	US	3.7	Q3
10	Journal of Intensive Care Medicine	30	US	3.1	Q3
11	Annals of Intensive Care	29	Germany	8.1	Q1

There are eight publishers headquartered in the United States, one in England, and one in Germany. The Cureus Journal of Medical Science, which is ranked 7th, does not have available information regarding its inclusion in the Chinese Academy of Sciences Journal Citation Reports or national ranking systems.

### Most cited publications

The prominence of research within a particular field is often demonstrated by the frequency of citations in scholarly publications. Table 2 presents a compilation of the ten most cited papers, revealing that the majority were published between 2004 and 2015, with 60% of them garnering over 1,000 citations. Notably, the paper with the highest number of citations is “Acute renal failure—definition, outcome measures, animal models, fluid therapy and information technology needs: the Second International Consensus Conference of the Acute Dialysis Quality Initiative (ADQI) Group,” which was published in 2004 (22). The paper titled “Surviving Sepsis Campaign: International guidelines for management of severe sepsis and septic shock: 2008,” published in Critical Care Medicine in 2008, ranks as the second most frequently referenced publication (23).

**Table 2:** The top 10 cited publications.

**Rank**	**Title**	**Year, Journal**	**First author**	**Total citations**
1	Acute renal failure – definition, outcome measures, animal models, fluid therapy and information technology needs: the Second International Consensus Conference of the Acute Dialysis Quality Initiative (ADQI) Group	2004, Critical Care	Rinaldo Bellomo	4,956
2	Surviving Sepsis Campaign: International guidelines for management of severe sepsis and septic shock: 2008	2008, Critical Care Medicine	Dellinger, R	4,084
3	Surviving Sepsis Campaign: International Guidelines for Management of Severe Sepsis and Septic Shock, 2012	2013, Critical Care Medicine	Dellinger, R	2,366
4	Surviving Sepsis Campaign guidelines for management of severe sepsis and septic shock	2004, Critical Care Medicine	Dellinger, R	2,101
5	Trial of Early, Goal-Directed Resuscitation for Septic Shock	2015, new england journal of medicine	Paul R. Mouncey	1,039
6	Fluid resuscitation in septic shock: A positive fluid balance and elevated central venous pressure are associated with increased mortality	2011, Critical Care Medicine	Boyd, John H	1,010
7	Sepsis: a roadmap for future research	2015, The Lancet Infectious Diseases	Jonathan Cohen	713
8	Guidelines for the diagnosis and management of disseminated intravascular coagulation	2009, British Journal of Haematology	M Levi	699
9	A positive fluid balance is associated with a worse outcome in patients with acute renal failure	2008, Critical Care	Didier Payen	689
10	Acute lung injury and the acute respiratory distress syndrome: a clinical review	2007, The Lancet	P Wheeler	663

### Citation burst analysis of references

**Figure 5:**
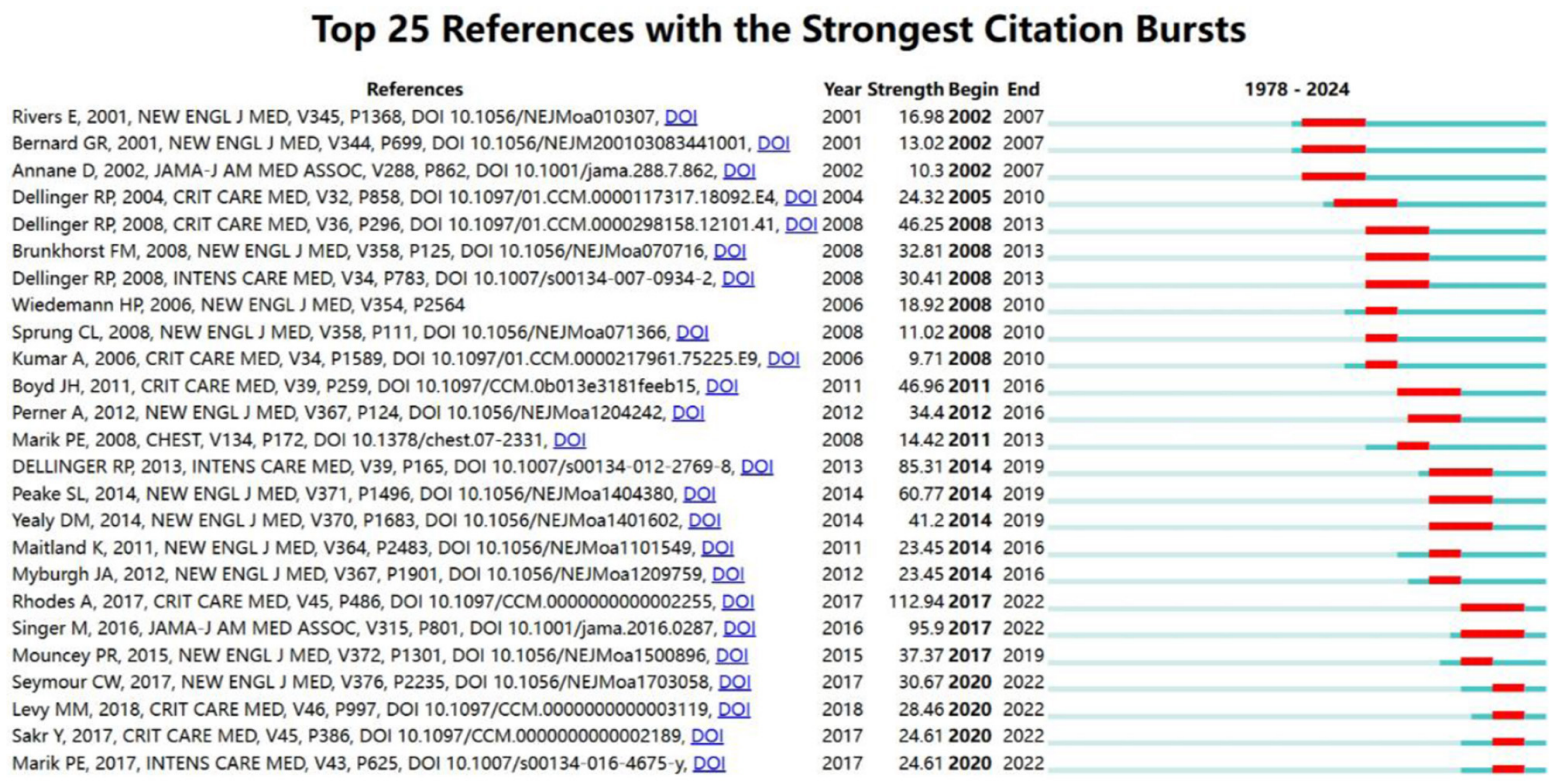
Top 25 cited references with the strongest citation bursts on tumor burden and immunotherapy.

Figure 5 displays the 25 most frequently cited references, with the dark blue line representing the citation frequency from 1978 to 2024 and the red line indicating the burst range of citation frequency, which has a minimum duration of 2 years.

The most cited reference with the highest burst value is the article by Rhodes titled “Surviving Sepsis Campaign: International Guidelines for Management of Sepsis and Septic Shock: 2016” (burst = 112.94, 2017–2022) (3). The second most cited reference is the article by Dellinger RP titled “Surviving Sepsis Campaign: International Guidelines for Management of Severe Sepsis and Septic Shock, 2012” (burst = 85.31) (13).

Between 2020 and 2022, four articles showed continuous citation bursts, with the highest burst value of 30.67 from the reference “Time to Treatment and Mortality during Mandated Emergency Care for Sepsis” (24). Among the recent burst references, the second most popular is “The Surviving Sepsis Campaign Bundle: 2018 Update” (25).

This analysis illuminates notable increases in citation frequency, suggesting periods of increased scholarly attention toward specific publications in the field of sepsis and fluid management.

### Keyword occurrence and co-occurrence analysis

**Figure 6:**
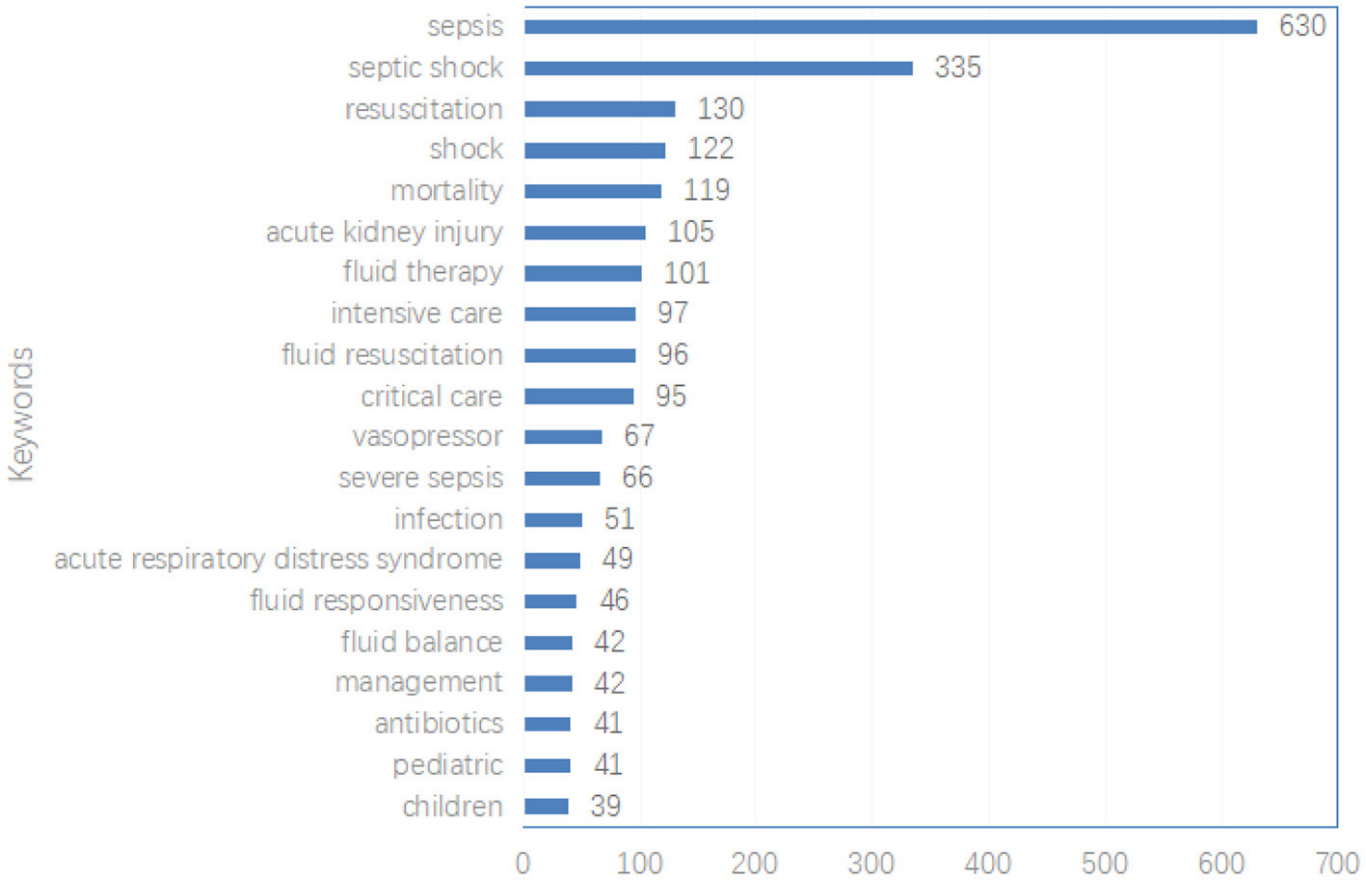
The top 20 most used keywords.

**Figure 7:**
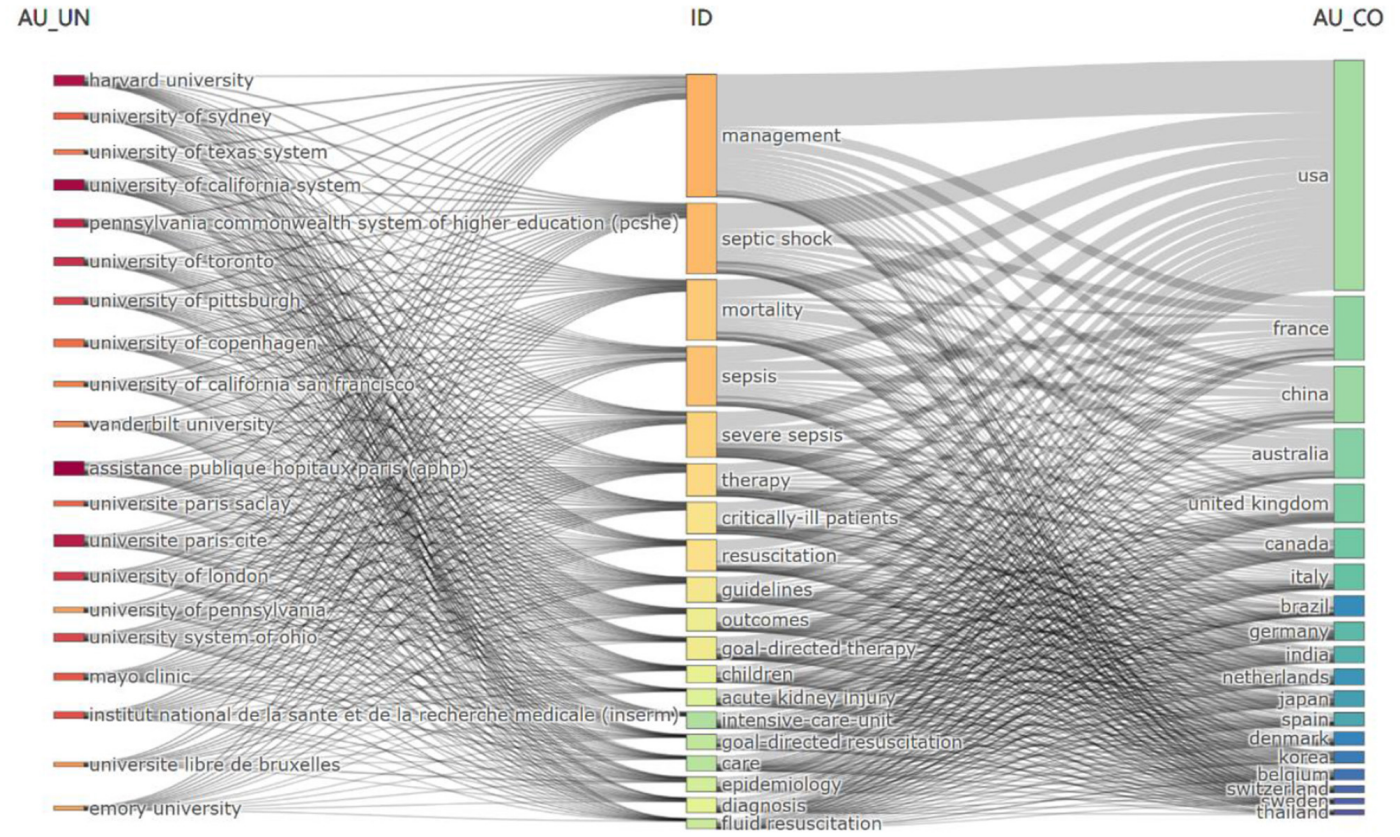
Three-field plot of the keywords plus analysis on Fluid Management of Sepsis (Left field: institutions; Middle field: keywords; Right field: countries).

This study analyzed a total of 4,177 keywords related to sepsis and fluid management. Figure 6 ranks the top 20 keywords by frequency, with “sepsis” appearing most frequently (630 occurrences), followed by “septic shock” (335 occurrences) and “resuscitation” (130 occurrences). Figure 7 further maps out the distribution of these keywords across institutions and countries, highlighting their associations with core topics in the field.

Nearly all institutions and countries contributed to the 19 main topics represented by these keywords, with prominent contributions from the United States and France at the institutional level.

### Co-occurrence analysis

**Figure 8:**
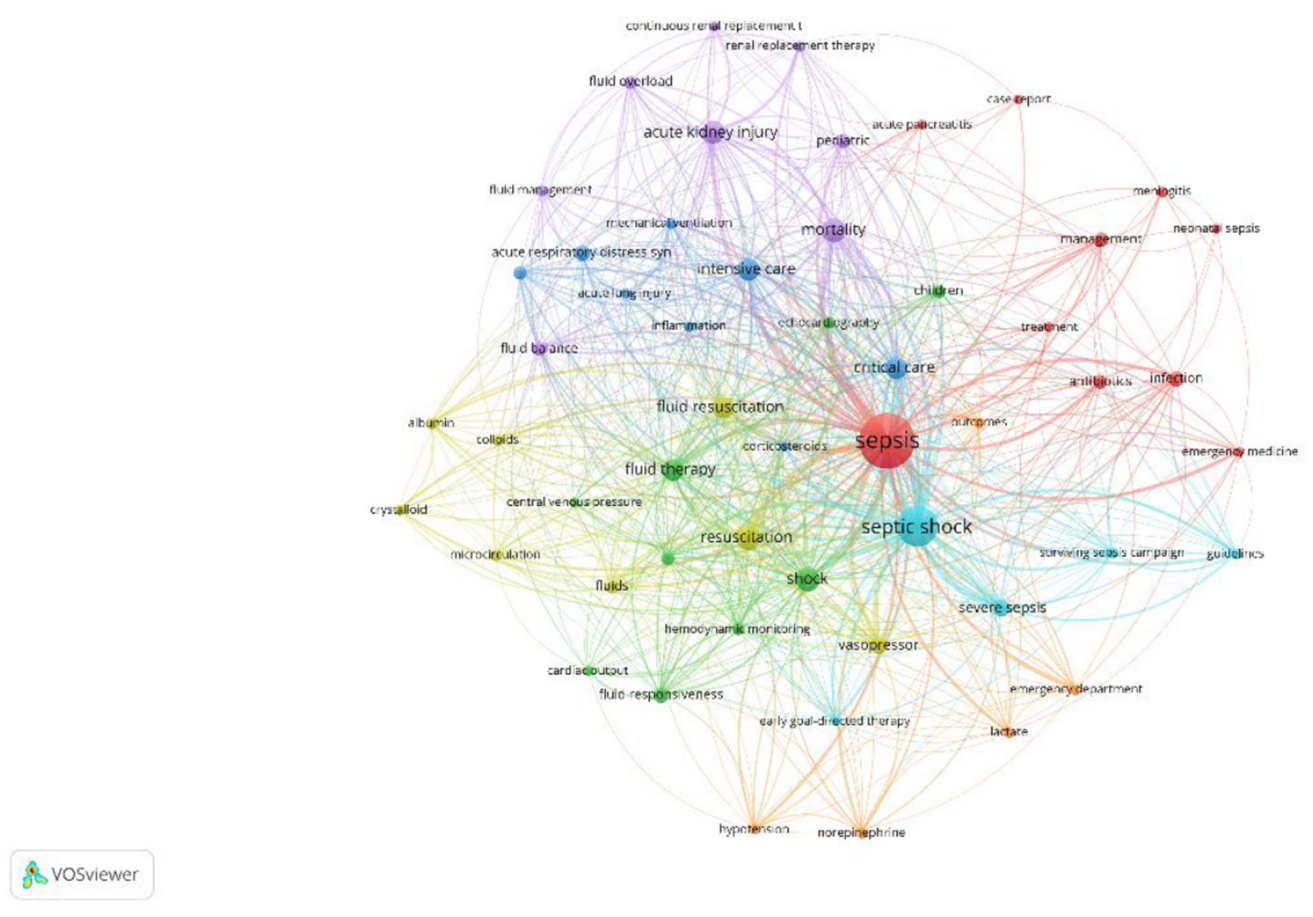
Keyword co-occurrence network.

Figure 8 presents a network graph of 53 keywords based on their co-occurrence patterns. Nodes are sized by keyword frequency and colored to indicate clusters of related keywords. Stronger relationships between keywords are denoted by shorter distances between nodes.

- **Cluster 1 (Red, 10 keywords)**: Focuses on sepsis-related diseases and treatments, including “sepsis,” “neonatal sepsis,” and “acute pancreatitis.”
- **Cluster 2 (Green, 9 keywords)**: Centers on indicators and methods related to fluid management, such as “central venous pressure,” “fluid therapy,” and “hemodynamic monitoring.”
- **Cluster 3 (Blue, 8 keywords)**: Primarily addresses critical care and intensive treatment topics like “critical care,” “intensive care,” and interventions such as “corticosteroids.”
- **Cluster 4 (Yellow, 8 keywords)**: Focuses on specific treatments like “albumin,” “colloid,” and “vasopressor.”
- **Cluster 5 (Purple, 8 keywords)**: Centers on outcomes and therapies related to mortality and fluid dynamics.
- **Cluster 6 (Light Blue, 5 keywords)**: Includes terms like “early goal-directed therapy” and “surviving sepsis campaign.”
- **Cluster 7 (Orange, 5 keywords)**: Discusses emergency department procedures and outcomes.

**Figure 9:**
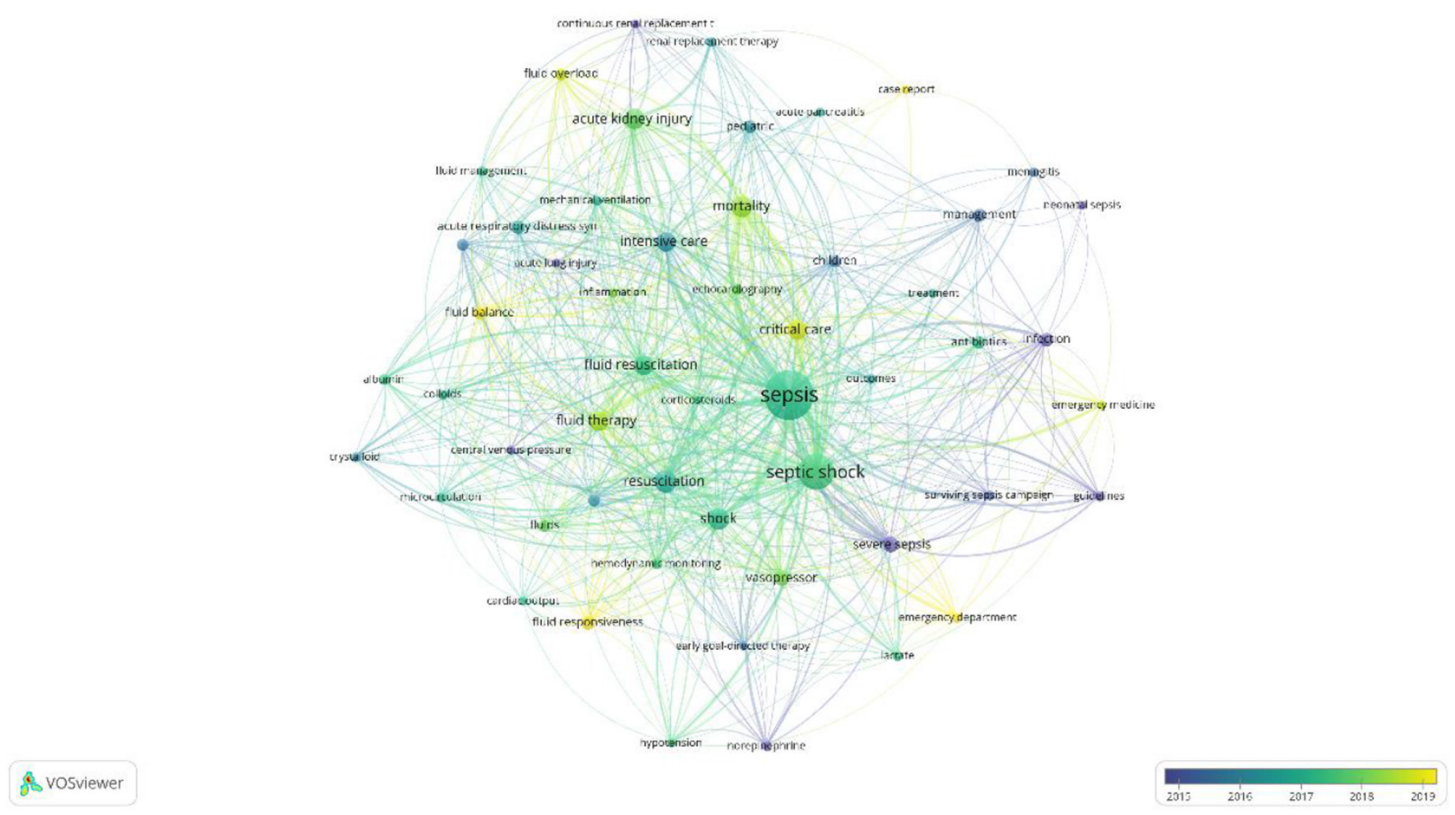
Keyword co-occurrence plus time-overlapping network.

Figure 9 demonstrates a temporal analysis of the co-occurring keywords, showcasing the evolution of research interests over time. Earlier research primarily concentrated on the management of severe sepsis and specific campaigns, whereas recent studies have shown a growing interest in areas such as fluid balance and emergency department protocols.

**Figure 10:**
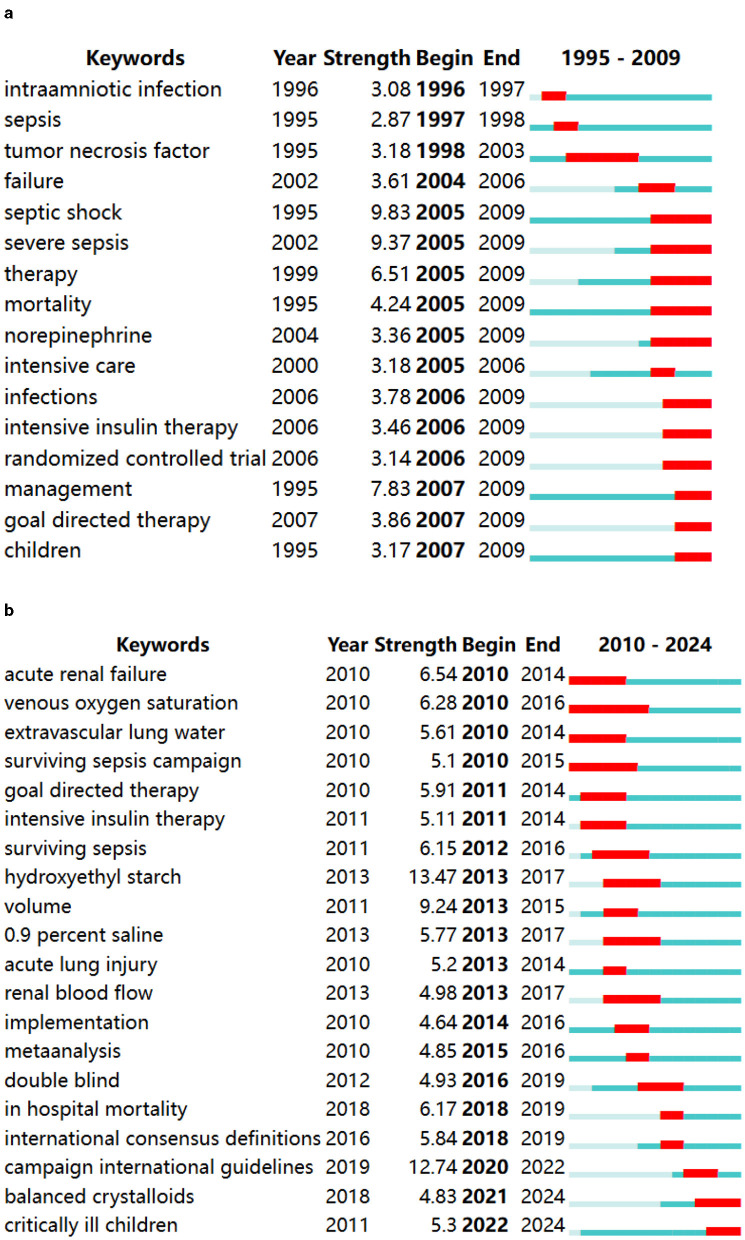
**(A)** The top 15 keywords with robust citation bursts in 1995–2009. **(B)** The top 20 keywords with robust citation bursts in 1910–2024.

The keyword burst analysis in Figures 10A, B presents the 16 strongest burst keywords from 1995 to 2009 and the 20 strongest burst keywords from 2010 to 2024, while no burst keywords were identified for the period from 1978 to 1994. From 1995 to 2009, “intraamniotic infection” and “sepsis” began to gain attention, gradually shifting over time to “septic shock,” “severe sepsis,” “therapy,” and “mortality.” Finally, “infections,” “intensive insulin therapy,” and “management” have been consistently focused on since their emergence. From 2010 to 2024, “acute renal failure,” “venous oxygen saturation,” “extravascular lung water,” and “surviving sepsis campaign” have all received continuous attention since their introduction. Additionally, “goal-directed therapy” was consistently highlighted from 2006 to 2014, while “balanced crystalloids” and “critically ill children” have gained increased attention recently, indicating that these keywords represent hot research topics in recent years and likely in the near future.

## Discussion

A bibliometric analysis was conducted on the literature pertaining to fluid therapy for sepsis patients from 1978 to June 2024. The initial publication on this topic dates back to 1978, authored by Tilney et al. Their findings underscored the importance of minimizing immunosuppressive measures to mitigate sepsis risk, as well as the efficacy of short-term antibiotic use in preventing wound infections during and after surgical procedures with potential contamination or sepsis (26).

### Trends in the number of publications

According to the yearly publication count, trends in publication growth can be categorized into periods of slow and rapid expansion. The slow growth phase spanned from 1978 to 2008, during which fewer than 50 articles were published annually. However, in 2008, there was a significant increase in the annual publication output of research on fluid therapy for sepsis patients. Subsequently, from 2009 to 2023, related research entered a period of rapid growth, with over 60 papers being published each year. This indicates that the field of fluid therapy for septic patients is likely to continue to experience significant activity and advancement in the foreseeable future. The Survival Sepsis Campaign Guidelines (SSCG) recommend aggressive fluid resuscitation in the early stages of treatment for severe sepsis and septic shock (27). The increasing focus and support from institutions on providing precise and efficient fluid management for sepsis patients have contributed to the notable growth rate observed in recent years (28).

The United States holds a prominent position in the field of fluid management in sepsis patients, as evidenced by its substantial publication output and significant role in international collaborations. The nation's leadership in this area serves as a benchmark on a global scale, reflecting not only its economic prowess but also its substantial investments in healthcare. Continued support for the national economy and international partnerships will further advance the comprehensive development of this research field.

The United States leads in the number of publications, with the top 10 countries collectively contributing 92.57% of total publications, and 9 of the top 15 institutions being American. Despite China ranking second in publications, none of its institutions are among the top 15. In contrast, the United Kingdom, ranking third in publishing output, has one institution in the top 15. Notably, UNIVERSITE LIBRE DE BRUXELLES and UNIVERSITY OF PITTSBURGH have been particularly active in recent years in terms of degrees awarded. Enhancing research competitiveness is contingent upon international collaboration, underscoring the critical necessity of fostering extensive partnerships among institutions, particularly in the face of economic constraints or resource limitations.

### Journal influence

When evaluating journal impact, indicators such as influence factor and JCR are significant measures (29, 30). Among the top 11 journals in the field of fluid management research for patients with sepsis, 36.4% are classified as JCR Q1 journals, with 6 of them having published over 1,000 papers. Critical Care and Critical Care Medicine stand out as the journals with the highest number of publications in this area. Core journals play a crucial role in disseminating fundamental research findings, making them ideal choices for researchers to submit their work.

### Research hotspots

This research aims to investigate the prevalent scientific emphasis on fluid management in septic patients within the academic community. Analysis of research trends can be conducted by examining publications, references, and keywords.

### Citation research hotspots

The frequency of citations for a publication can serve as a metric for assessing its impact within a particular field of study (31). Frequently cited works often represent central themes in research, aiding in the identification of key areas of focus. Among the top 10 most cited papers are those addressing topics such as Surviving Sepsis Campaign—International Guidelines for the Management of Severe Sepsis and Septic Shock (23), Guidelines for the Management of Severe Sepsis and Septic Shock within the Surviving Sepsis Campaign (27), Early Goal-Directed Therapy Trial for Septic Shock (32), and Fluid Resuscitation for Septic Shock (33).

In the first version of the international consensus definition from 1991, severe sepsis was defined as sepsis accompanied by organ dysfunction, hypoperfusion, or hypotension; septic shock was defined as sepsis with hypotension despite adequate fluid resuscitation. In 2016, following the publication of the third edition of the international consensus definitions for sepsis and septic shock (Sepsis-3), the definition of sepsis was updated. This consensus recommended that organ dysfunction should be defined according to the Sequential (or Sepsis-related) Organ Failure Assessment (SOFA) score or the “quick” (q) SOFA. Septic shock was also redefined as a subset of sepsis, characterized by persistent hypotension requiring vasopressor therapy to maintain a mean arterial pressure ≥65 mmHg, along with a serum lactate level >2 mmol/L (>18 mg/dL). Septic shock indicates severe circulatory, cellular, and metabolic deterioration, with a higher risk of mortality compared to simple sepsis (1).

Patients with severe sepsis frequently exhibit varying degrees of cognitive impairment, as well as a dysregulated systemic inflammatory response that results in widespread vasodilation and increased capillary permeability. This leads to significant alterations in vascular distribution, reduced effective circulating volume, decreased ventricular preload, diminished diastolic pressure, decreased cardiac output, and impaired oxygen delivery. In response to these changes, the body initiates a cascade of cardiovascular, neuroendocrine, and metabolic adaptations to compensate for the loss of blood volume. If the injury factors persist or the inflammatory response remains uncontrolled, the body may exhibit decompensation manifestations, including impairment of endothelial cell function and dysfunction of microcirculation, leading to systemic tissue hypoxia, organ dysfunction, and potentially mortality.

Liquid therapy has the potential to enhance microcirculation perfusion through mechanisms such as lowering blood viscosity, augmenting blood flow driving pressure, and modulating the interplay between endothelial cells and circulating blood cells. Furthermore, liquid therapy has been shown to substantially diminish the concentrations of inflammatory mediators TNF-α, IL-8, and IL-1B triggered by endotoxins, while concurrently elevating levels of the anti-inflammatory mediator IL-10.The peak impact is observed 1–6 h following the initiation of the inflammatory response (34). A retrospective cohort study carried out by Alsous et al. (35) demonstrated that patients with septic shock who sustained a negative fluid balance for a minimum of 1 day within 72 h of initial intensive care unit (ICU) treatment exhibited a more favorable prognosis.

Vincent and De Backer introduced a theoretical framework for fluid management in the management of septic shock patients, delineating the treatment process into four distinct phases: resuscitation, optimization, stability, and descent (36). The resuscitation phase aims to attain a viable minimum blood pressure level essential for sustaining life, while the optimization phase focuses on enhancing cardiac output to meet the body's anticipated demands. The stable phase emphasizes organ support and complication prevention, while the descent phase involves a gradual reduction of intensive care unit interventions for patients. The model also highlights the importance of fluid resuscitation therapy. Malbrain examined various fluid management strategies, such as early adequate goal-directed fluid management, late conservative fluid management, and late goal-directed fluid removal (37). The authors also introduced the “4D” framework for fluid therapy, encompassing drug selection, dosage determination, treatment duration, and de-escalation strategies. In the management of patients with septic shock, it is essential to consider the four phases of fluid therapy, including the initiation of intravenous infusion, cessation of intravenous infusion, commencement of reverse resuscitation or fluid removal, and discontinuation of reverse resuscitation in order to achieve the objectives of each stage.

Dellinger et al. (27) introduced the Surviving Sepsis Campaign: International Guidelines for Management of Severe Sepsis and Septic Shock, which were subsequently revised in 2008, 2012, 2016, and 2021 (3, 12, 13, 23, 27). The guidelines recommend programmed and quantitative resuscitation for patients experiencing tissue hypoperfusion due to sepsis, characterized by sustained hypotension following initial rapid fluid replacement or a blood lactate concentration of ≥4 mmol/L. Upon identification of tissue hypoperfusion, immediate intervention is recommended rather than waiting until the patient is transferred to the intensive care unit (ICU). Within the first 6 h of early recovery from hypoperfusion due to sepsis, treatment goals include maintaining a central venous pressure (CVP) of 8–12 mm Hg, a mean arterial pressure (MAP) of ≥65 mm Hg, a urine output of ≥0.5 ml · kg-1 · h-1, and achieving a central venous oxygen saturation (ScvO2) of ≥0.70 or mixed venous oxygen saturation (SvO2) of ≥0.65.Crystalloid solution is the preferred initial resuscitation treatment for severe sepsis and septic shock in liquid therapy. In cases where a significant volume of crystalloid fluid is required for fluid resuscitation, the use of albumin is recommended. In instances where there is suspicion of hypoperfusion in sepsis with low blood volume, a minimum of 30 ml/kg of crystalloid solution (potentially including albumin) is advised for shock therapy. Certain patients may necessitate more rapid and substantial fluid replacement.

The sudden analysis feature in CiteSpace is utilized to identify references and keywords that have experienced significant changes during specific time periods. Strength, as an indicator in the analysis of citation bursts for references and keywords, is a measure of their attractiveness and has been extensively examined in the literature. Additionally, the timing of citation explosions provides insights into the duration of citations and the current level of attention received in the field. It also provides an aspect of research hotspots. In this study, “guidelines,” “sepsis,” “rescue,” and “outcome” were the keywords that most recently exploded until 2024. They all appeared in time overlap analysis before. For references, there have been 6 papers cited since 2017, and this explosion continued until 2022. This study explores research hotspots by analyzing the keywords “guidelines,” “sepsis,” “rescue,” and “outcome” that have recently gained prominence until 2024. These keywords were identified through time overlap analysis and have been cited in 6 papers since 2017, with this trend continuing until 2022. One of the cited papers discusses the definition and treatment management guidelines for sepsis, including The Third International Consensus Definitions for Sepsis and Septic Shock (Sepsis-3) and Surviving Sepsis Campaign: International Guidelines for Management of Sepsis and Septic Shock: 2016 (1). Two scholarly articles discuss fluid therapy for sepsis, with one focusing on the expedited completion of the 3-h sepsis treatment and rapid administration, albeit facing challenges in promptly completing the initial intravenous infusion (15), while the other delves into the effectiveness and prognosis of bundle therapy for sepsis patients (19). A study showed that the average fluid volume administered to patients with severe sepsis and septic shock on the 1^st^ day in the ICU was lower than the fluid amounts recommended by the Surviving Sepsis Campaign guidelines. Administering more than 5L of fluid on the 1^st^ day in the ICU was associated with a significantly increased risk of death and significantly higher hospital costs (38).

Through the analysis of citations, it can be observed that continuous exploration of sepsis management and ongoing comparison of research outcomes have led to corresponding modifications in the Surviving Sepsis Campaign from 2004 to 2021. This ensures that the most favorable recommendations for sepsis treatment are provided. Additionally, concepts such as bundled therapy and goal-directed fluid management have also been proposed in the guidelines, which is why the guidelines remain the most cited literature in recent years.

### Keyword research hotspots

Keywords are essential elements that encapsulate the main themes of a research study, with their frequency serving as a measure of their significance within particular academic domains. These keywords also serve to highlight prominent areas of research interest, with prevalent terms in the field of “liquid management” including “rescue,” “fluid therapy,” “fluid rescue,” “fluid responsiveness,” “fluid balance,” and “management.” The keywords associated with a cluster of diseases encompass “acute kidney injury,” “septic shock,” and “acute respiratory distress syndrome,” along with the medications “vasopressor” and “antibiotics.”

This study analyzes the research hotspots in sepsis fluid management over a 15-year interval. It was found that no research hotspots emerged between 1978 and 1994, likely due to insufficient publication of literature during this period, as sepsis fluid management was still in an exploratory phase with few results published. Starting from 1998, articles on sepsis fluid management began to be published, discussing fluid management from various perspectives, including intraamniotic infection, tumor necrosis factor, and mortality. In 2001, sepsis was refined into septic shock and severe sepsis based on severity, leading to detailed fluid management for different types of sepsis, and the concept of early goal-directed therapy (EGDT) was introduced for the first time. Subsequently, goal-directed therapy became a research hotspot. “Acute renal failure” emerged as the first burst keyword from 2010 to 2024. During this period, researchers focused on indicators such as “venous oxygen saturation,” “volume,” and “renal blood flow.” Additionally, “balanced crystalloids” and “critically ill children” represent hot research topics in recent years and likely in the near future.

It is worth noting that the “Campaign International Guidelines” in 2019 had a strength value of 12.74 for the period 2019–2022. In 2019, COVID-19 spread rapidly worldwide, triggering an unprecedented public health crisis. The clinical manifestations of many severe or critically ill COVID-19 patients meet the diagnostic criteria for sepsis and septic shock. Studies have shown that the dysregulation of the innate immune response caused by SARS-CoV-2 leads to a high inflammatory syndrome that exacerbates disease severity and increases mortality (39, 40). Severe COVID-19 patients may develop sepsis, disseminated intravascular coagulation, and multiple organ dysfunction. Therefore, for severe COVID-19 patients, in addition to treating the primary disease, attention to sepsis-related fluid management is also crucial. After 2022, due to the continuous mutations of the COVID-19 virus and the widespread use of vaccines, the number of severe COVID-19 patients has decreased, leading to a decline in research interest regarding the treatment of severe COVID-19.

Early goal-directed therapy (EGDT) has undoubtedly been a milestone in the treatment of severe sepsis and septic shock, with fluid therapy being a crucial component of EGDT (41). This approach compared a standard treatment group with a proactive resuscitation strategy that included aggressive intravenous fluid administration, vasopressor use, and blood transfusions. In the standard treatment group, patients underwent arterial and central venous catheter placement, with intravenous fluids administered to maintain a central venous pressure of 8–12 mmHg and vasopressors used to keep mean arterial pressure >65 mmHg. The early goal-directed therapy group had the same hemodynamic targets but also received continuous monitoring of central venous oxygen saturation, using dobutamine to achieve a target central venous saturation of ≥70%. Within the first 6 h of intervention, the early goal-directed therapy group received more intravenous fluids, blood transfusions, and did receive a higher percentage of dobutamine, along with lower lactate levels. Compared to the standard treatment group, the early goal-directed therapy group showed a 16% reduction in in-hospital mortality. This study reflected that the bundled approach to fluid resuscitation via early goal-directed therapy significantly improves the prognosis of patients with septic shock, leading to its inclusion in early sepsis management guidelines. A randomized controlled trial further demonstrated that EGDT aims to restore hemodynamic stability and tissue perfusion indicators in patients with severe sepsis and septic shock, emphasizing early identification of those with tissue hypoperfusion and implementing a series of interventions guided by specific monitoring indicators within 6 h. The findings indicated that EGDT significantly reduces in-hospital mortality in patients with severe sepsis and septic shock, shortens hospital stays, and decreases healthcare costs (42). The timing of fluid therapy is critical for the prognosis of patients with severe sepsis. Early fluid treatment can modulate the inflammatory response and improve microvascular perfusion, thereby positively impacting organ function and patient outcomes. However, in the later stages of sepsis, fluid therapy has limited effects on tissue perfusion, and excessive fluid overload could worsen patient prognosis (43).

“Acute renal failure” is the first burst keyword from 2010 to 2024.Murphy and colleagues conducted an analysis to assess the effects of various liquid therapy approaches on in-hospital mortality among patients with severe sepsis complicated by acute lung injury (ALI) (44). The liquid therapy strategies under investigation included adequate initial fluid resuscitation (AIFR) and conservative late fluid management (CLFM). In the present study, AIFR was operationally defined as an infusion volume equal to or >20 ml/kg prior to the administration of vasoconstrictors, with ongoing fluid replacement to achieve a central venous pressure (CVP) of at least 8 mmHg within 6 h following the initiation of vasoconstrictors. The definition of CLFM entails the maintenance of a stable or negative fluid balance within the initial 7 days following the onset of septic shock, as evidenced by consistent inflow and outflow rates for a minimum of 2 consecutive days. The findings of the study indicate that the timing of fluid therapy in sepsis plays a crucial role in influencing the in-hospital mortality rate of patients with septic shock complicated by acute lung injury (ALI). As such, it is recommended that tailored fluid therapy strategies be implemented for patients with severe sepsis and septic shock, taking into consideration the individual's disease progression and underlying pathophysiological features. Specifically, early aggressive fluid resuscitation followed by a more conservative approach later in the treatment course may be beneficial.

In 2016, the “Sepsis Campaign” guidelines recommended an aggressive fluid resuscitation method for patients with septic shock, which is considered a cornerstone of treatment. This involves infusing at least 30 mL/kg of crystalloid solution for initial resuscitation within the first 3 h of shock onset (3). While this is a strong recommendation, the quality of evidence supporting it is considered low. By 2021, the same fluid resuscitation protocol was downgraded from a strong recommendation to a weak recommendation in the “Sepsis Campaign” guidelines, indicating that the dosage of fluid resuscitation remains a hot topic of debate in septic shock management (12). The purpose of initial fluid resuscitation is to restore the patient's vascular volume, increase cardiac output, enhance oxygen delivery, and improve tissue oxygenation. Therefore, the “1-h bundle strategy” emphasizes that fluid resuscitation in septic patients must be initiated within 1 h to promptly correct the pathophysiological processes caused by sepsis, including reduced effective circulating volume and impaired microcirculation perfusion (3, 37). A study by Tseng et al. showed that balanced crystalloids and albumin are more effective than hydroxyethyl starch and normal saline in reducing mortality among septic patients (45). Another study found that septic shock patients who received more fluids within the initial 3 h were more likely to survive. In septic shock, increased vasodilation and vascular permeability lead to relative and absolute hypovolemia, making the goal of initial fluid resuscitation to restore blood volume, thereby increasing cardiac output and oxygen delivery (46). Rapid and substantial initial fluid resuscitation can improve microcirculation and tissue perfusion to some extent, thereby enhancing prognosis, including lowering SOFA scores, reducing hospital stays, and decreasing mortality rates (47). Therefore, optimizing fluid resuscitation strategies remains a crucial aspect of improving outcomes for patients with septic shock (48).

Despite studies indicating that aggressive resuscitation does not increase mortality rates, positive fluid balance, and its duration have been shown to be associated with higher mortality (39). Conservative resuscitation can achieve fluid negative balance more rapidly. In the treatment of septic patients, rapid and large-volume fluid administration can lead to the dangers of fluid overload, causing patient suffering, adverse reactions, and increased economic burden. Late-stage sepsis may present with elevated central venous oxygen saturation and lactate levels; at this point, even if adequate resuscitation is performed, it may not improve microcirculatory perfusion. Therefore, conservative resuscitation aims to reduce the volume of fluid administered during prolonged resuscitation, initiate vasopressors early, and provide the minimum fluid necessary to maintain circulation.

The development of sepsis fluid management has gradually evolved from the classification of the disease to the refined management of indicators. It has progressed from bundled care and goal-directed therapy for fluid resuscitation in the early stages of the disease to corresponding crystalloid balance or volume balance based on indicator monitoring in the later stages, avoiding excessive load. This approach combines the use of vasopressors and antimicrobial treatment to promote patient recovery. Therefore, in the later stages, sepsis fluid management tends to focus on indicator monitoring and control.

In the near future, research interests may focus on the following two topics: (1) The efficacy and prognosis of fluid management regimens for sepsis patients, including staged fluid management and bundled management; (2) Explore the underlying mechanisms of the relationship between fluid load and septic shock treatment.

### Limitations

It is important to recognize that this study is subject to various limitations. Primarily, being a bibliometric analysis, the collection and processing of data heavily rely on software. While this analysis cannot entirely supplant system retrieval, it does enable a thorough examination of extensive data sets. Additionally, it is important to note that this study exclusively utilized English articles sourced from the Web of Science database, potentially excluding valuable research. However, given the extensive coverage of the Web of Science in scholarly literature, it is anticipated that any oversights will not substantially affect the overarching trends identified in the study. Third, due to citation impact delays, some high-quality studies published in recent years may not have had the opportunity to showcase their valuable breakthroughs due to low burst value, such as balanced crystalloids and critically ill children, requiring tracking and updates in future research.

Nevertheless, considering the comprehensive scope of the Web of Science in academic publications, it is expected that any potential oversights will not significantly impact the overarching patterns identified in the study.

## Conclusion

In conclusion, there is a growing focus on the connection between fluid management and the treatment of sepsis, with research in this area being at an advanced stage. The most noteworthy new clues that people are paying attention to in future research hotspots are: (1) The efficacy and prognosis of liquid management schemes for sepsis patients, including staged liquid management and bundled management; (2) Explore the underlying mechanisms of the relationship between fluid load and septic shock treatment. The analysis results indicate that researchers have conducted more accurate and in-depth research in the field of fluid management for sepsis patients.

## Data Availability

The original contributions presented in the study are included in the article, further inquiries can be directed to the corresponding authors.

## References

[1] Singer M Deutschman CS Seymour CW Shankar-Hari M Annane D Bauer M . The third international consensus definitions for sepsis and septic shock (Sepsis-3). JAMA. (2016) 315:801–10. 10.1001/jama.2016.0287 26903338 PMC4968574

[2] Huang M Cai S Su J. The pathogenesis of sepsis and potential therapeutic targets. Int J Mol Sci. (2019) 20:5376. 10.3390/ijms20215376 31671729 PMC6862039

[3] Rhodes A Evans LE Alhazzani W Levy MM Antonelli M Ferrer R . Surviving sepsis campaign: international guidelines for management of sepsis and septic shock: 2016. Intensive Care Med. (2017) 43:304–77. 10.1007/s00134-017-4683-6 28101605

[4] Fleischmann-Struzek C Mellhammar L Rose N Cassini A Rudd KE Schlattmann P . Incidence and mortality of hospital- and ICU-treated sepsis: results from an updated and expanded systematic review and meta-analysis. Intensive Care Med. (2020) 46:1552–1562. 10.1007/s00134-020-06151-x 32572531 PMC7381468

[5] Liu V Escobar GJ Greene JD Soule J Whippy A Angus DC . Hospital deaths in patients with sepsis from 2 independent cohorts. Jama. (2014) 312:90–2. 10.1001/jama.2014.5804 24838355

[6] Fleischmann C Scherag A Adhikari NK Hartog CS Tsaganos T Schlattmann P . Assessment of global incidence and mortality of hospital-treated sepsis. Current estimates and limitations. Am J Respir Crit Care Med. (2016) 193:259–72. 10.1164/rccm.201504-0781OC 26414292

[7] Jawad I Luksic I Rafnsson SB. Assessing available information on the burden of sepsis: global estimates of incidence, prevalence and mortality. J Glob Health. (2012) 2:010404. 10.7189/jogh.01.010404 23198133 PMC3484761

[8] Brown RM Semler MW. Fluid management in sepsis. J Intensive Care Med. (2019) 34:364–73. 10.1177/0885066618784861 29986619 PMC6532631

[9] Milford EM Reade MC. Resuscitation fluid choices to preserve the endothelial glycocalyx. Crit Care. (2019) 23:77. 10.1186/s13054-019-2369-x 30850020 PMC6408751

[10] Dhondup T Tien J-CC Marquez A Kennedy CC Gajic O Kashani KB. Association of negative fluid balance during the de-escalation phase of sepsis management with mortality: a cohort study. J Crit Care. (2020) 55:16–21. 10.1016/j.jcrc.2019.09.025 31670149

[11] Besen BAMP Taniguchi LU. Negative fluid balance in sepsis: when and how? Shock. (2016) 47:1. 10.1097/SHK.0000000000000701 27454378

[12] Evans L Rhodes A Alhazzani W Antonelli M Coopersmith CM French C . Surviving sepsis campaign: international guidelines for management of sepsis and septic shock 2021. Crit Care Med. (2021) 49:e1063–143. 10.1007/s00134-021-06506-y 34605781

[13] Dellinger RP Levy MM Rhodes A Annane D Gerlach H Opal SM . Surviving sepsis campaign: international guidelines for management of severe sepsis and septic shock, 2012. Intensive Care Med. (2013) 39:165–228. 10.1007/s00134-012-2769-8 23361625 PMC7095153

[14] Acheampong A Vincent JL. A positive fluid balance is an independent prognostic factor in patients with sepsis. Crit Care. (2015) 19:251. 10.1186/s13054-015-0970-1 26073560 PMC4479078

[15] Dabi Y Darrigues L Katsahian S Azoulay D De Antonio M Lazzati A. Publication trends in bariatric surgery: a bibliometric study. Obes Surg. (2016) 26:2691–9. 10.1007/s11695-016-2160-x 27052317

[16] Ozsoy Z Demir E. The evolution of bariatric surgery publications and global productivity: a bibliometric analysis. Obes Surg. (2018) 28:1117–29. 10.1007/s11695-017-2982-1 29086169

[17] Avcu G Bal ZS Duyu M Akkus E Karapinar B Vardar F. Thanks to trauma: a delayed diagnosis of pott disease. Pediatr Emerg Care. (2015) 31:e17–8. 10.1097/PEC.0000000000000637 26626903

[18] van Eck NJ Waltman L. Citation-based clustering of publications using CitNetExplorer and VOSviewer. Scientometrics. (2017) 111:1053–70. 10.1007/s11192-017-2300-7 28490825 PMC5400793

[19] van Eck NJ Waltman L. Software survey: VOSviewer, a computer program for bibliometric mapping. Scientometrics. (2010) 84:523–38. 10.1007/s11192-009-0146-3 20585380 PMC2883932

[20] Chen C. Searching for intellectual turning points: progressive knowledge domain visualization. Proc Natl Acad Sci U S A. (2004) 101 Suppl 1:5303–10. 10.1073/pnas.0307513100 14724295 PMC387312

[21] Yao L Hui L Yang Z Chen X Xiao A. Freshwater microplastics pollution: detecting and visualizing emerging trends based on Citespace II. Chemosphere. (2020) 245:125627. 10.1016/j.chemosphere.2019.125627 31864046

[22] Bellomo R Ronco C Kellum JA Mehta RL Palevsky P workgroup ADQI. Acute renal failure - definition, outcome measures, animal models, fluid therapy and information technology needs: the Second International Consensus Conference of the Acute Dialysis Quality Initiative (ADQI) Group. Crit Care. (2004) 8:R204–12. 10.1186/cc2872 15312219 PMC522841

[23] Dellinger RP Levy MM Carlet JM Bion J Parker MM Jaeschke R . Surviving Sepsis Campaign: international guidelines for management of severe sepsis and septic shock: 2008. Crit Care Med. (2008) 36:296–327. 10.1097/01.CCM.0000298158.12101.41 18158437

[24] Seymour CW Gesten F Prescott HC Friedrich ME Iwashyna TJ Phillips GS . Time to treatment and mortality during mandated emergency care for sepsis. New England J Med. (2017) 376:2235–44. 10.1056/NEJMoa1703058 28528569 PMC5538258

[25] Levy MM Evans LE Rhodes A. The surviving sepsis campaign bundle: 2018 update. Intensive Care Med. (2018) 44:925–8. 10.1007/s00134-018-5085-0 29675566

[26] Tilney NL Strom TB Vineyard GC Merrill JP. Factors contributing to the declining mortality rate in renal transplantation. N Engl J Med. (1978) 299:1321. 10.1056/NEJM197812142992401 101845

[27] Dellinger RP Carlet JM Masur H Gerlach H Calandra T Cohen J . Surviving Sepsis Campaign guidelines for management of severe sepsis and septic shock. Crit Care Med. (2004) 32:858–73. 10.1097/01.CCM.0000117317.18092.E4 15090974

[28] Jain RK Antonio BL Bowton DL Houle TT MacGregor DA. Variability in central venous pressure measurements and the potential impact on fluid management. Shock. (2010) 33:253–7. 10.1097/SHK.0b013e3181b2bb22 19543151

[29] Zimmerman J Field J Leusch F Lowry GV Wang P Westerhoff P. Impact beyond impact factor. Environ Sci Technol. (2022) 56:11909. 10.1021/acs.est.2c05553 35984216

[30] Atallah AN Puga M Amaral J. Web of Science Journal Citation Report 2020: the Brazilian contribution to the “Medicine, General & Internal” category of the journal impact factor (JIF) ranking (SCI 2019). Sao Paulo Med J. (2020) 138:271–4. 10.1590/1516-3180.2020.138419092020 33111803 PMC9673832

[31] Aksnes DW Langfeldt L Wouters P. Citations, citation indicators, and research quality: an overview of basic concepts and theories. SAGE Open. (2019) 9:215824401982957. 10.1177/2158244019829575

[32] Mouncey PR Osborn TM Power GS Harrison DA Sadique MZ Grieve RD . Trial of early, goal-directed resuscitation for septic shock. N Engl J Med. (2015) 372:1301–11. 10.1056/NEJMoa1500896 25776532

[33] Boyd JH Forbes J Nakada TA Walley KR Russell JA. Fluid resuscitation in septic shock: a positive fluid balance and elevated central venous pressure are associated with increased mortality. Crit Care Med. (2011) 39:259–65. 10.1097/CCM.0b013e3181feeb15 20975548

[34] Dorresteijn MJ van Eijk LT Netea MG Smits P van der Hoeven JG Pickkers P. Iso-osmolar prehydration shifts the cytokine response towards a more anti-inflammatory balance in human endotoxemia. J Endotoxin Res. (2005) 11:287–93. 10.1179/096805105X58715 16263001

[35] Alsous F Khamiees M DeGirolamo A Amoateng-Adjepong Y Manthous CA. Negative fluid balance predicts survival in patients with septic shock: a retrospective pilot study. Chest. (2000) 117:1749–54. 10.1378/chest.117.6.1749 10858412

[36] Vincent JL De Backer D. Circulatory shock. N Engl J Med. (2013) 369:1726–34. 10.1056/NEJMra1208943 24171518

[37] Malbrain MLNG Van Regenmortel N Saugel B De Tavernier B Van Gaal P-J Joannes-Boyau O . Principles of fluid management and stewardship in septic shock: it is time to consider the four D's and the four phases of fluid therapy. Ann Intensive Care. (2018) 8:66. 10.1186/s13613-018-0402-x 29789983 PMC5964054

[38] Marik PE Linde-Zwirble WT Bittner EA Sahatjian J Hansell D. Fluid administration in severe sepsis and septic shock, patterns and outcomes: an analysis of a large national database. Intensive Care Med. (2017) 43:625–32. 10.1007/s00134-016-4675-y 28130687

[39] Arina P Singer M. Pathophysiology of sepsis. Curr Opin Anaesthesiol. (2021) 34:77–84. 10.1097/ACO.0000000000000963 33652454

[40] Xu Z Shi L Wang Y Zhang J Huang L Zhang C . Pathological findings of COVID-19 associated with acute respiratory distress syndrome. Lancet Respir Med. (2020) 8:420–2. 10.1016/S2213-2600(20)30076-X 32085846 PMC7164771

[41] Rivers E Nguyen B Havstad S Ressler J Muzzin A Knoblich B . Early goal-directed therapy in the treatment of severe sepsis and septic shock. N Engl J Med. (2001) 345:1368–77. 10.1056/NEJMoa010307 11794169

[42] Bozza FA Carnevale R Japiassú AM Castro-Faria-Neto HC Angus DC Salluh JI. Early fluid resuscitation in sepsis: evidence and perspectives. Shock. (2010) 34:40–3. 10.1097/SHK.0b013e3181e7e668 20714265

[43] Bouchard J Soroko SB Chertow GM Himmelfarb J Ikizler TA Paganini EP . Fluid accumulation, survival and recovery of kidney function in critically ill patients with acute kidney injury. Kidney Int. (2009) 76:422–7. 10.1038/ki.2009.159 19436332

[44] Murphy CV Schramm GE Doherty JA Reichley RM Gajic O Afessa B . The importance of fluid management in acute lung injury secondary to septic shock. Chest. (2009) 136:102–9. 10.1378/chest.08-2706 19318675

[45] Tseng C-H Chen T-T Wu M-Y Chan M-C Shih M-C Tu Y-K. Resuscitation fluid types in sepsis, surgical, and trauma patients: a systematic review and sequential network meta-analyses. Crit Care. (2020) 24:693. 10.1186/s13054-020-03419-y 33317590 PMC7734863

[46] Lee SJ Ramar K Park JG Gajic O Li G Kashyap R. Increased fluid administration in the first three hours of sepsis resuscitation is associated with reduced mortality: a retrospective cohort study. Chest. (2014) 146:908–15. 10.1378/chest.13-2702 24853382 PMC4188147

[47] Self WH Semler MW Bellomo R Brown SM deBoisblanc BP Exline MC . Liberal versus restrictive intravenous fluid therapy for early septic shock: rationale for a randomized trial. Ann Emer Med. (2018) 2018:S0196064418303159. 29753517 10.1016/j.annemergmed.2018.03.039 PMC6380679

[48] Leisman DE Doerfler ME Schneider SM Masick KD D'Amore JA D'Angelo JK. Predictors, prevalence, and outcomes of early crystalloid responsiveness among initially hypotensive patients with sepsis and septic shock. Crit Care Med. (2018) 46:189–98. 10.1097/CCM.0000000000002834 29112081

